# Cuticular Barcoding—A Novel Method for Host Identification From Adult Parasitoid Wasps

**DOI:** 10.1002/ece3.74529

**Published:** 2026-10-18

**Authors:** Maura Haas‐Renninger, Michael Haas, Manuel Stothut, Henrik Krehenwinkel, Johannes L. M. Steidle, Lars Krogmann

**Affiliations:** ^1^ State Museum of Natural History Stuttgart Stuttgart Germany; ^2^ Center of Excellence for Biodiversity and Integrative Taxonomy (KomBioTa) Stuttgart Germany; ^3^ Adelaide University Adelaide South Australia Australia; ^4^ Department of Biogeography Trier University Trier Germany; ^5^ University of Hohenheim, Institute of Biology Stuttgart Germany

**Keywords:** biological interactions, microhymenoptera, molecular method, natural enemies, species networks

## Abstract

Parasitoid wasps are important regulators of their hosts and highly relevant for the resilience of terrestrial ecosystems. However, data on parasitoid‐host interactions are scarce and missing for many parasitoid species as parasitoids are mostly collected as adults without host association and rearing of parasitoids from their hosts can be difficult. Molecular methods can thus help to circumvent this bottleneck. We here present cuticular barcoding, a novel non‐destructive molecular approach to identify parasitoid‐host associations from adult parasitoid wasps. We tested our approach by rearing the parasitoid wasp *Lariophagus distinguendus* on two beetle species, and washing off remnant host DNA from the wasp cuticle to detect the host species using DNA barcoding. Our results showed a success rate of 49%, detecting the host species in 35 out of 72 samples. Samples containing pooled wasp specimens performed equally well as single wasp specimens, which indicates that single field‐collected wasps are sufficient to identify host associations. Hosts could be detected in wasps that were fixated either through freezing or ethanol, which also supports the application of this method in the field. The primers Jerry/Pat targeting the mitochondrial COI gene performed better than the universal primers LCO/HCO, indicating that more studies testing universal primers are needed to establish this method. This non‐destructive method allows to preserve the sampled wasp as a physical voucher for subsequent morphological identification which will be essential for its usage in situ. Our study presents a novel method that enables detection of parasitoid‐host interactions, and holds significant potential for conservation ecologists, pest managers and evolutionary biologists.

## Graphical abstract

Our manuscript presents a novel molecular tool that permits host detection of an adult parasitoid wasp. Our non‐destructive method, that we here tested in a laboratory experiment, allows studying the intricate interactions between parasitoids and their hosts while preserving the parasitoid as voucher specimen for subsequent morphological identification. With further development, it can facilitate the study of parasitoid‐host interactions in the field, laying the baseline for creating interaction networks.

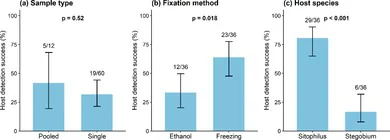

## 1 Introduction

In times of global biodiversity crisis, understanding the complex interplay between species and their environment is key to taking measures to conserve biodiversity. Especially insects, which contribute to ecosystem functioning through varied interactions like pollination, host regulation, and nutrient recycling, are decreasing on species and population level (Hallmann et al. 2017; Seibold et al. 2019) due to habitat loss through intensive farming, use of pesticides, and climate change (Basset et al. 2017; Rumohr et al. 2023; Sánchez‐Bayo and Wyckhuys 2019). Within the insect class, Hymenoptera (sawflies, wasps, bees, and ants) is supposed to be the most species‐rich order of all, as it mostly comprises parasitoid wasps that are mostly specialized on their hosts (Forbes et al. 2018). However, the diversity of parasitoid Hymenoptera (including ‘Parasitica’ and aculeates with a parasitoid lifestyle) and their role in ecosystems is hard to grasp, as they are highly understudied due to their predominantly small size and the lack of taxonomic resources (Hartop et al. 2022; Hausmann et al. 2020; Shaw and Hochberg 2001). Even for described species, host records and knowledge on life histories are often scarce (Moser et al. 2024). One reason for this is that rearing parasitoid wasps from their hosts can be very time‐consuming, especially for small‐sized wasps (microhymenoptera) that parasitize endophytic hosts living in plant structures or host eggs which are very small and thus hard to collect in the field. In addition, parasitoids are mostly collected as adults with sweep nets or Malaise traps without information on the association to their hosts. Considering the fundamental role of parasitoids in regulating their host populations as natural enemies and contributing to the resilience of terrestrial ecosystems (LaSalle and Gauld 1991), there is a need for an easy and time‐efficient method to better understand the bipartite interaction of microhymenoptera and their hosts.

There were several studies that used molecular approaches, such as DNA barcoding, to assess parasitoid‐host interactions to build ecological networks (Evans et al. 2016), also in the context of pest control (Derocles et al. 2014; Gariepy et al. 2007; Henneman and Memmott 2001; Traugott and Symondson 2008). For these studies, hosts were actively collected and used for molecular analysis using tissue samples (Hrček et al. 2011; Šigut et al. 2017; Traugott and Symondson 2008; Wolz et al. 2024) or gut content of adult parasitoid wasps (Rougerie et al. 2011; Wirta et al. 2014). In this context, metabarcoding and next‐generation sequencing seem promising in providing cost‐efficient approaches for detecting trophic interactions (Kitson et al. 2018; Lefort et al. 2017). Although these approaches detected a higher number of trophic links compared to traditional rearing (Wirta et al. 2014; Kitson et al. 2018), they are faced with limitations. First, they focus on parasitoids of hosts that can be easily collected in the field and second, studies on gut content result in the destruction of the wasp specimens, making it impossible to preserve physical vouchers for morphological identification. However, after molting into an imago, insects possess a rigid exoskeleton that can be used to study their interactions with the environment during their most mobile phase, including their developmental host, using eDNA. Birer et al. (2017) studied the bacterial community on the cuticle of ants, providing a protocol to wash off bacterial DNA from the ants, enabling detection of low DNA quantity and preserving the integrity of the ant specimens. This protocol has a high potential for studying other Hymenoptera groups, such as parasitoid wasps, and their interactions with their hosts. The parasitoid wasp *Lariophagus distinguendus* (Foerster) (Hymenoptera: Pteromalidae) is a solitary ectoparasitic idiobiont that attacks hosts that are concealed in seeds, grain kernels, or cocoons (Kaschef 1959; Hagstrum and Subramanyam 2009). Various aspects of its biology have been studied in the past, and it is nowadays commercially used as a biological control agent against a wide variety of material and storage pests like the granary weevil *Sitophilus granarius* (Linnaeus) (Coleoptera: Curculionidae) (Niedermayer et al. 2016). Due to its accessibility and easy handling in the laboratory, it is ideally suited as a model organism to test novel molecular methods for the detection of parasitoid‐host interactions.

In this paper, we introduce “cuticular barcoding” as a novel molecular approach to determine parasitoid‐host interactions based on washing adult parasitoid wasps to detect the host species from which they emerged. In a laboratory experiment, we tested the hypothesis that remnant host DNA on the cuticle of the adult parasitoid wasp can be used to identify the host species using DNA barcoding. We therefore used *Lariophagus distinguendus* wasps that had been lab‐reared on either larvae of *S. granarius* or *Stegobium paniceum* (Linnaeus) (Coleoptera: Ptinidae) to detect their host species by washing the parasitoid wasps using the protocol provided by Birer et al. (2017), and barcoding the collected DNA. This method has the potential to revolutionize the study of parasitoid‐host interactions by enabling the identification of host species of parasitoid wasps without the destruction of the specimens and thus advancing our understanding of parasitoid‐host interaction networks.

## 2 Material and Methods

### 2.1 Specimen Collection

*L. distinguendus* wasps were obtained from the Department of Chemical Ecology of the University of Hohenheim and were reared from one of two host species: *S. granarius* grown on wheat grains (*Tritium aetivum* L.) and *St. paniceum* grown on koi pellets (Hikari Friend, Kamihata Fish Industry Group, Kyorin Corporation, Japan). For our analysis, we used wasps that recently emerged from their hosts. Single grains and pellets containing host larvae were placed in 1.5 mL Eppendorf tubes and incubated in a rearing chamber with an ambient temperature of 25.5°C and a humidity of 45%–50%. Tubes were controlled twice a day, in the morning and in the afternoon. Emerged wasps were sexed, transferred to a new, sterile Eppendorf tube, and either frozen at −20°C or preserved in 99.6% pure ethanol. In total, we analyzed 109 wasps (78 females; 31 males) reared from *S. granarius* and 69 wasps (49 females; 20 males) reared from *St. paniceum*.

### 2.2 Molecular Processing

#### 2.2.1 DNA Extraction (Wasp Washing)

As we expected host DNA to be present in very small quantities, we kept contamination risk in the laboratory as low as possible by using sterile tools and equipment and surfaces cleaned with RNase AWAY (Thermo Fisher Scientific, Waltham, MA, USA) and 70% ethanol. Host DNA was washed from the wasp cuticle using an adapted protocol of Birer et al. (2017). We added 200 μL Buffer A per wasp specimen and vortexed for 1 min. The buffer was then transferred to a new sterile 1.5 mL tube for DNA extraction. 99% pure ethanol was added to the wasp specimens with subsequent storage at −20°C for further analysis. We used a QIAamp DNA Mini Kit (QIAamp; Qiagen, Carlsbad, CA, USA) for extraction of DNA, as the smaller pore size of this kit limits DNA loss compared to other kits (Birer et al. 2017). Lysis time was set to 60 min and the recommended Step 8 for risk reduction of buffer carryover was performed. We extracted three specimens of each host species, *S. granarius* and *St. paniceum* by destructive sampling using the whole body of the specimens to generate a reference barcode and to test the viability of different primers. We extracted cuticular DNA from 60 single wasp specimens (30 from *S. granarius* and 30 from *St. paniceum*) and 12 specimen pools (6 pools per species). Each pool consisted of 5 wasp specimens (3 females and 2 males). Within each host species, samples were equally allocated to the two preservation methods (freezing or ethanol). Thus, each host species was represented by 15 individual wasp specimens and 3 pools per preservation method. A detailed distribution of samples is provided in Table 1.

**TABLE 1:** Sample distribution per host species and preservation method. Number of female (f) and male (m) wasps is shown in brackets.

Species	Preservation method	Single specimens	Pools	Specimens per pool	Total specimen number
*Sitophilus granarius*	ethanol 99.6%	15 (10f:5 m)	3	5 (3f:2 m)	30
*Sitophilus granarius*	freezing −20°C	15 (10f:5 m)	3	5 (3f:2 m)	30
*Stegobium paniceum*	ethanol 99.6%	15 (10f:5 m)	3	5 (3f:2 m)	30
*Stegobium paniceum*	freezing −20°C	15 (10f:5 m)	3	5 (3f:2 m)	30

#### 2.2.2 PCR Amplification and Sequencing

To test if host DNA can be amplified from the parasitoid wasp cuticle, we tested four different primer pairs targeting the mitochondrial COI gene for amplifying DNA of the two beetle species using the DNA extracted directly from the beetle specimens (Table 2). Based on the gel band intensity, LCO1490/HCO2198 and Jerry/Pat were determined to yield the best results and were therefore subsequently used in the amplification of the eluated cuticular DNA from the washed wasps. For PCR amplification the FastGene Optima HotStart ReadyMix (Nippon Genetics, Düren, Germany) was used with a total of 25 μL reaction volume, including 12.5 μL Taq Ready Mix, 6 μL H_2_O, 1.25 μL 10 μM primer forward, 1.25 μL 10 μM primer reverse and 4 μL of DNA eluate. The thermocylcer protocol was set up as follows: lid temperature of 110°C, initial denaturation at 94°C for 2 min; followed by 5 cycles of denaturation at 96°C for 30 s, annealing at 45°C for 1 min and elongation at 72°C for 1 min; followed by 35 cycles of denaturation at 93°C for 1 min, annealing at 50°C for 1 min and elongation at 72°C for 1 min; with a final elongation step at 72°C for 5 min. PCR products producing strong bands after gel electrophoresis were sent to Eurofins Genomics Europe Shared Services GmbH (Ebersberg, Germany) for purification and standard bidirectional Sanger‐sequencing.

**TABLE 2:** Primers used for amplification.

Primer	Direction	Primer sequence 5–3	Amplicon length	References
LCO1490	F	GGTCAACAAATCATAAAGATATTGG	658	Folmer et al. 1994
HCO2198	R	TAAACTTCAGGGTGACCAAAAAATCA	658	Folmer et al. 1994
LepF1	F	ATTCAACCAATCATAAAGATATTGG	658	Hajibabaei et al. 2006
LepR1	R	TAAACTTCTGGATGTCCAAAAAATCA	658	Hajibabaei et al. 2006
Ron	F	GGATCACCTGATATAGCATTCCC	440	Simon et al. 1994
Tom	R	ACRTAATGAAARTGGGCTACWA	440	Ribera et al. 2010
Jerry	F	CAACATTTATTTTGATTTTTTGG	830	Simon et al. 1994
Pat	R	TCCAATGCACTAATCTGCCATATTA	830	Simon et al. 1994

#### 2.2.3 Sequence Analysis

We used the forward and reverse reads including the corresponding chromatogram files provided by the sequencing company to assemble consensus sequences to enable quality control of base calls, trimming of low‐quality regions, and manual inspection of ambiguous positions or discrepancies between forward and reverse reads. Raw Sanger reads of the primer pairs LCO1490/HCO2198 and Jerry/Pat were de novo assembled with Geneious version 10.2.6 (Kearse et al. 2012) under the “Highest Sensitivity/Slow” setting. Primer regions were cut and the consensus sequence was generated. Host identification via the cuticular DNA was performed by comparing the generated consensus sequences against the previously assembled sequences of *St. paniceum* and *S. granarius* . Sequences failing host identification were blasted through NCBI services (Sayers et al. 2022) to obtain further identity information.

#### 2.2.4 Statistical Analysis

Statistical analysis was performed using R version 4.4.1 (R Core Team 2021) and RStudio version 2024.04.2 (Posit team 2023). We defined success rate as the proportion of samples in which the host species was successfully detected compared to the total number of samples. Each pooled sample was treated as one experimental unit. Fisher's exact test and Pearson's Chi squared test were used for pairwise comparisons between groups. For graphical presentation, 95% Wilson confidence intervals were calculated for each proportion.

## 3 Results

### 3.1 Primer Testing

Our primer tests showed that all the tested primer pairs were able to target *S. granarius* and *St. paniceum*. However, the primer pairs LCO1490/HCO2198 and Jerry/Pat showed the strongest bands in the gel‐electrophoresis, which is why we used these two primer pairs for the detection of the host from the wasp cuticle for a total of 72 samples, consisting of 36 wasps reared from *S. granarius* and 36 wasps reared from *St. paniceum*.

### 3.2 Host Detection

We extracted 72 wasp samples and were able to identify their host in 35 samples (49%). These samples contained pooled samples with several wasp specimens as well as single wasp specimens. There was no difference in the detection rate between these two categories (*p* = 0.52, Fisher's Exact Test; Figure 1). For the single individuals, sex did not have an effect on detection success (*χ* ^2^ = 1.01, df = 1, *p* = 0.315). The comparison of the fixation methods showed that freezing led to a significantly higher number of host detections compared to ethanol as a fixation agent (*χ* ^2^ = 5.56, df = 1, *p* = 0.018, Figure 1). The primers Jerry/Pat successfully amplified correct host DNA in a total of 35 samples. The standard DNA barcoding primers LCO1490/HCO2198 only amplified host DNA in 13 samples, all of which were also amplified by the primers Jerry/Pat. Samples of wasps that were reared on *S. granarius* showed a higher correct amplification rate than those reared on *St. paniceum* (*χ* ^2^ = 26.91, df = 1, *p* < 0.001, Figure 1). In 23 samples, the parasitoid wasp *L. distinguendus* was amplified. Contamination was very low with two samples from *S. granarius* containing DNA from either *Wolbachia* sp. or from *Homo sapiens* .

**FIGURE 1:**
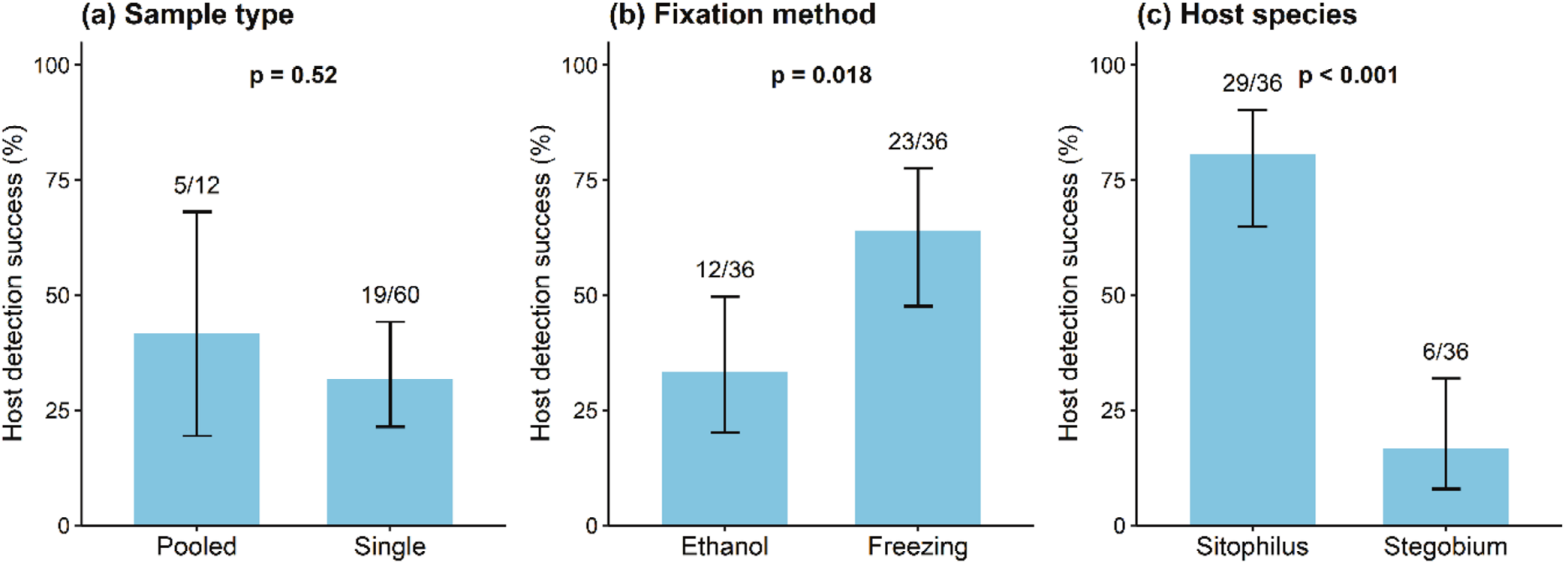
Host detection success based on DNA of washed *Lariophagus distinguendus* samples dependent on (a) sample type, (b) fixation method, and (c) host species from which wasps were reared. Error bars represent 95% Wilson confidence intervals. Labels indicate the number of host‐positive samples relative to the total number of independent samples (positive/total). Each pooled sample consisted of five wasps and was treated as one independent sample.

## 4 Discussion

Our study reveals that remnant host DNA on the cuticle of an adult parasitoid wasp can be used to identify its host species using DNA barcoding. Interacting organisms leave traces of DNA which are already known from eDNA metabarcoding (Banerjee et al. 2022; Ruppert et al. 2019; Taberlet et al. 2018), where whole insect communities can be assessed even from washing off plant leaves (Stothut et al. 2024). Due to its biology as an ectoparasitic idiobiont, *L. distinguendus* is in close contact with its host during its development. In the larval stage, it is crawling and feeding on the host from the outside. After molting into an adult, the wasp loads detectable host DNA on its cuticle when it gets into contact with host remains in the confined inner space of the food kernel.

Our tested primer pairs showed different results in their performance. Jerry/Pat detected the beetle hosts more often than LCO1490/HCO2198, which is reasonable since Jerry/Pat are used mainly for Coleoptera. For using cuticular barcoding in the field, universal primers and primer cocktails should be tested as the purpose of this method is to detect the so‐far unknown host species of a parasitoid wasp.

The detection success of pooled specimens in our study was not significantly higher than that of single specimens, which might have been due to the lower sample size in the pooled group compared to single individuals. However, our results clearly show that a single wasp specimen may be sufficient to identify its host species. Detection success from wasps that were frozen was higher than from wasps fixated in ethanol. Our results show that wasps collected in ethanol can still be used to reliably detect the host species, which is highly important for using cuticular barcoding of field‐sampled wasps. We focused on freezing and high‐concentration ethanol as fixation methods, which might be challenging in the field. Conditions such as lower ethanol concentrations, room‐temperature storage, as well as long‐term preservation might have an effect on the detection success and should be tested in future studies. Detection success of wasps reared from *S. granarius* was higher than from *St. paniceum*. At present, we cannot provide an explanation for this finding, as both beetle species develop inside their food resource, forming a closed environment in which the parasitoid wasp is in direct contact with the host DNA. DNA decay rates may differ between species (Friedrich et al. 2024), but there is no literature on closely related species decay rates. Although measures to prevent contamination were applied, two samples were contaminated with *Wolbachia* and human DNA. *Wolbachia* are endosymbiotic bacteria that are known to occur in *S. granarius* (Tuncbilek et al. 2015) as well as in the lineage GWsat of *L. distinguendus* used in our experiments (König et al. 2019). This makes detection of *Wolbachia* in cuticular DNA extracts from *L. distinguendus* biologically plausible. Contamination with human DNA could have occurred prior to the DNA extraction during the rearing and specimen handling, highlighting the susceptibility of low‐biomass samples to exogenous DNA and the need for strict contamination‐control measures throughout DNA barcoding experiments.

We used the ectoparasitoid wasp species *L. distinguendus* reared on two beetle host species as a model organism to test whether it was generally possible to detect remnants of host DNA from the cuticle of the adult parasitoid wasp. Therefore, our results demonstrate the feasibility of cuticular barcoding under controlled laboratory conditions, but further investigations are needed to establish its general applicability across parasitoid taxa and biological settings, especially for application in the field. Detection success may differ among parasitoid species and host systems as host‐DNA transfer and persistence on the wasp cuticle may be influenced by various factors such as parasitoid developmental strategy, host biology, adult emergence behavior, and time after emergence. Endoparasitoids may represent another promising group for future applications as they develop inside their host where they are in closer host contact than ectoparasitoids. In the same way, the application to polyphagous parasitoids requires further investigation.

Metabarcoding could be beneficial for field‐collected specimens as it might be more sensitive for fragmented DNA. Under field conditions, parasitoid wasps might get in contact with DNA from non‐host organisms through direct interaction or passively through the environment which might lower the reliability of the results. Sequence read counts and relative read abundance in metabarcoding can give an idea of the time and frequency species interacted with each other (Friedrich et al. 2024), and might be indicative of the host species of a field‐collected wasp, but this would need to be experimentally tested. Further, environmental exposure might have an impact on host detection success as the host DNA might degrade by time through ambient temperature and UV radiation or washed off by rainfall (Deagle et al. 2006; Valentin et al. 2021). To account for these issues, smaller markers (mini‐barcodes) could be used instead of standard 658 bp barcodes as they might yield better results for fragmented DNA. In our study, we used a single set of conditions for host DNA elution as we were working under controlled laboratory conditions. In the future, the protocol could be optimized through gradient tests on the elution volume and elution times to capture even smaller DNA amounts. Further laboratory experiments would be needed to develop best practice approaches for conducting cuticular barcoding of field‐collected parasitoids. Despite these limitations, the overall host detection success of 49% observed in our study was comparatively high relative to the 24% reported previously for gut content analyses of parasitoids (Rougerie et al. 2011), particularly because we used standard DNA barcoding and Sanger‐sequencing. These results indicate that cuticular barcoding has potential as a molecular approach for investigating parasitoid‐host interactions.

Building parasitoid‐host interaction networks can be challenging as reliable data on species interactions are scarce especially for parasitoid‐host associations (Hrček and Godfray 2015). Furthermore, available data are biased towards larger sized individuals (Chase and Knight 2013). Thus, cuticular barcoding has enormous potential as it enables identifying the host of a parasitoid wasp that was sampled in the field without information on the association with its host. In addition, cuticular barcoding is a non‐destructive method, and the parasitoid wasp can be preserved as a physical voucher for subsequent verification via morphological identification. Working with adult parasitoid wasps also guarantees the host‐suitability of a detected host species as eggs laid in unsuitable hosts are likely to fail to develop (Condon et al. 2014). Using this molecular method in combination with traditional methods, such as rearing, has the potential to significantly increase our understanding of species interaction networks (Wirta et al. 2014). Cryptic interactions such as parasitoids of hidden eggs or larvae, or between “dark taxa” such as platygastrine wasps and gall midges (Awad et al. 2025), could be discovered more easily, and complex interaction networks involving hyperparasitoids could be disentangled. Multitrophic interaction networks could be studied by incubating a whole plant and performing cuticular barcoding on the emerging insects.

Cuticular barcoding could also be highly valuable to foster biological control as it would enable the discovery of potential natural enemies as control agents and could in the long term contribute to reducing the usage of pesticides. In addition, it could be used to test the efficacy of released natural enemies against pest species for classical biological control in agricultural contexts and may further reveal if the biological control agent uses non‐target host organisms which might have implications for local biodiversity. Reconstructing links between parasitoid wasps and their hosts and building parasitoid‐host interaction networks would also be highly relevant for conservation biologists maintaining viable ecosystems. Parasitoids are on a high trophic level and can be used in biodiversity monitoring as indicators for downstream diversity (Anderson et al. 2011). Thus, cuticular barcoding of parasitoid wasps can be a time‐ and cost‐effective approach for biodiversity monitoring as it may allow retrieval of a species interaction network from just a single specimen. Thereby, it may help to monitor the effects of landscape changes and conservation measures on species interaction networks without the need of intensive sampling for identification purposes. Finally, cuticular barcoding could be valuable for evolutionary biologists studying species interactions as a driver for diversification and speciation (Hambäck et al. 2024; Harmon et al. 2019; Hufbauer and Via 1999).

## 5 Conclusion

Our results for the first time show that it is possible to detect remnants of host DNA on the cuticle of an adult parasitoid wasp using DNA barcoding. Although this new method is highly promising for biodiversity monitoring, biological control and evolutionary biology, further studies are needed to assess the applicability of this method in the field. For example, its success rate should be investigated for different parasitoid life histories (endo‐ and ectoparasitoids), contamination with non‐host organisms, and environmental factors that can impact the DNA. We here used standard DNA barcoding, but in the future the protocol could be further developed for a metabarcoding approach which might yield better results in the field where DNA is more likely to be fragmented. If this method shows to be reliable for parasitoid‐host systems, its application may be expanded to other groups such as predator–prey systems.

## Author Contributions

**Maura Haas‐Renninger:** conceptualization (equal), data curation (equal), formal analysis (lead), investigation (equal), visualization (lead), writing – original draft (lead). **Michael Haas:** conceptualization (equal), data curation (equal), investigation (equal), methodology (lead), writing – original draft (equal). **Manuel Stothut:** methodology (supporting), validation (equal), writing – review and editing (equal). **Henrik Krehenwinkel:** methodology (supporting), validation (equal), writing – review and editing (equal). **Johannes L. M. Steidle:** validation (equal), writing – review and editing (equal). **Lars Krogmann:** funding acquisition (equal), writing – review and editing (equal).

## Funding

Funding for Maura Haas‐Renninger was provided by the Ministry of Science and Art of Baden‐Wuerttemberg.

## Conflicts of Interest

The authors declare no conflicts of interest.

## Data Availability

The data that support the findings of this study are available in dryad: https://doi.org/10.5061/dryad.5dv41nsp4.
